# CD133+ cells are associated with ADIPOCYTOKINES and endothelial dysfunction in hemodialysis patients

**DOI:** 10.1186/s12882-017-0663-x

**Published:** 2017-07-26

**Authors:** Abdullah Ozkok, Riza Atas, Suzan Adin Cinar, Akar Yilmaz, Esin Aktas, Gunnur Deniz, Alaattin Yildiz

**Affiliations:** 1 Department of Nephrology, Istanbul Medeniyet University, Goztepe Training and Research Hospital, Istanbul, Turkey; 2 Division of Medicine, Department of Cardiology, Kliniken Calw, Calw, Germany; 3 Depatment of Immunology, Istanbul University, Experimental Medical Research Institute, Istanbul, Turkey; 4 Department of Cardiology, Izmir University Hospital, Izmir, Turkey; 5 Department of Nephrology, Istanbul University, Istanbul Faculty of Medicine, Istanbul, Turkey

**Keywords:** CD133+ cells, Adipocytokines, Leptin, Adiponectin, Resistin, Tumor necrosis factor-α, Interleukin-6, Flow-mediated dilatation, Endothelial dysfunction, Atherosclerosis

## Abstract

**Background:**

Hemodialysis (HD) patients have increased risk of cardiovascular disease (CVD). Impaired stem cell health and adipocytokine metabolism may play important roles in the complex pathophysiological mechanisms of CVD in this patient population. We aimed to investigate the relationships between CD133+ cell counts, adipocytokines and parameters of endothelial dysfunction and atherosclerosis in HD patients.

**Methods:**

In 58 chronic HD patients (male/female:28/30, mean age:58 ± 14 years), serum levels of interleukin-6 (IL-6), tumor necrosis factor-α (TNF-α), leptin, adiponectin and resistin were measured by ELISA. Left ventricular mass index (LVMI), carotid intima-media thickness (CIMT), flow-mediated dilatation (FMD) of the brachial artery were measured. CD133+ cells were counted by flow cytometry (BD FACSCalibur-BD Bioscience,CA).

**Results:**

CD133+ cell counts were inversely associated with FMD (*r* = −0.39, *p* = 0.007) and positively correlated with serum resistin (*r* = 0.45, *p* < 0.001) and serum TNF-α (*r* = 0.31, *p* = 0.02). Serum leptin levels were higher in high CD133 group compared to low CD133 group [32.37(12.74–72.29) vs 15.50(5.38–37.12)ng/mL, *p* = 0.03]. Serum leptin levels were correlated with TNF-α(*r* = 0.35, *p* = 0.009). Serum adiponectin levels were negatively correlated with serum leptin (*r* = −0.28, *p* = 0.03). Serum resistin levels were associated with TNF-α (*r* = 0.54, *p* < 0.001) and leptin (*r* = 0.29, *p* = 0.03). Serum IL-6 levels were significantly associated with LVMI (*r* = 0.31, *p* = 0.03). Serum IL-6 levels were significantly higher in patients with carotid plaque compared to patients without plaque [12.75(9.91–28.68) vs 8.27(5.97–14.04) pg/mL, *p* = 0.02]. In multiple linear regression analysis to determine the factors predicting LogFMD; dialysis vintage, LVMI and LogCD133+ cell counts were included as independent variables(*R* = 0.57, adjusted R-square = 0.27, *p* = 0.001). CD133+ cell count and LVMI were found to significantly predict FMD (*p* = 0.03 and *p* = 0.04 respectively).

**Conclusion:**

CD133+ cells were associated with inflammation and endothelial dysfunction in HD patients. Serum leptin, resistin and TNF-α levels were positively related to CD133+ cell count. Impaired regulation of undifferentiated stem cells and adipocytokines might contribute to endothelial dysfunction in HD patients.

## Background

Increased CV risk in CKD patients is not explained completely with the conventional CV risk factors [1, 2] and new pathophysiological mechanisms such as impaired stem cell turnover might be responsible for dramatically increased CV mortality in this patient population. In general, circulating stem cells are known to have pro-angiogenic and repair properties in various tissues. CD133+ identifies a subset of undifferentiated stem cells which have been investigated for pathogenesis and prevention of CV diseases in several studies [3–5]. In the study by Stamm et al. [4], purified CD133+ progenitor cells were injected in the infarct border zone during the coronary artery bypass grafting operation in patients with chronic ischemic heart disease and ejection fraction was found to be higher in this cell therapy group. In another study, CD133+ cell administration was found to reduce cortical infarct volumes in mice following cerebral ischemia [5]. However, in several other studies, CD133+ cells were found to have possible detrimental effects such as increased inflammation, atherosclerotic plaque instability and progression of atherosclerosis [6–10]. In a study by Pizarro et al. [10], CD45+/CD34+/CD133+ cells were found to be inversely associated with flow mediated dilatation (FMD) in patients with chronic obstructive pulmonary disease (COPD). All these studies were performed on non-CKD population. In the literature, there is only one study investigating the possible effect of CD133+ cells on a CV parameter in CKD patients. In this study performed on HD patients, CD133+ cell count were not found to be associated with left ventricular mass index (LVMI) [11].

Adipocytokines may also contribute to increased CV risk in CKD patients. Adipocytokines such as leptin, adiponectin, resistin, interleukin-6 (IL-6) and tumor necrosis factor-α (TNF-α) are mediators produced by adipocytes which have important roles in the pathophysiology of insulin resistance, inflammation, hypertension, endothelial dysfunction and atherosclerosis [12–18]. In this study, we aimed to investigate the relationships between CD133+ cell counts, adipocytokines and parameters of endothelial dysfunction and atherosclerosis in HD patients.

## Subjects and methods

Fifty-eight chronic HD patients (male/female: 28/30, mean age: 58 ± 14 years) were included. We included the chronic HD patients who had been on HD for more than 6 months between the ages 18–85. Exclusion criteria were as follows, having a recent acute CV event within 6 months of period, advanced heart failure, decline of the consent and unsuitable upper extremities for FMD measurements. Mean time on HD was 81.6 ± 44.4 months. Patients were dialyzed three times a week for 4 h per session, using blood flow rates of 350–400 ml/min and dialysate flows of 500 ml/min. All patients were dialyzed with a standard bicarbonate-containing dialysate bath. Etiologies of CKD were as follows: hypertension, 14 (24.1%); diabetes mellitus, 6 (10.3%); chronic glomerulonephritis, 10 (17.2%); others, 6 (10.3%) and unknown in 22 patients (37.9%).

All fasting blood samples were collected before the midweek HD session in the study group and serum samples were obtained and stored at -80 °C. Laboratory values including complete blood counts and serum levels of urea nitrogen, creatinine, electrolytes, calcium, phosphorus, total protein, albumin, total cholesterol and triglycerides were measured. High sensitive C-reactive protein (hsCRP) was measured by nephelometric method (Dade Behring, Marburg, Germany). This study was done in accordance with the ethical principles of the Declaration of Helsinki. Written informed consents were obtained from all patients. This study was approved by the Ethical Committee of Istanbul Faculty of Medicine (2009/1859).

### ELISA measurements

Serum levels of IL-6 (Invitrogen, CA, USA), TNF-α (Invitrogen, CA, USA), leptin (Linco Research, St. Charles, MO, USA), adiponectin (B-bridge International, USA), resistin (Biovendor, Czech Republic) were measured by the enzyme-linked immunoadsorbent assays (ELISA) methods.

### Echocardiography

Echocardiography was performed in the day after HD (Vingmed System Five echocardiographic system, Norway). Both M-Mode and 2D measurements were performed according to the methods recommended by the American Society of Echocardiography. Left ventricular hypertrophy (LVH) was defined as LVMI >134 g/m^2^ for males and >110 g/m^2^ for females. Left ventricular mass was calculated using the formula: 1.04 (Diastolic LV diameter + septal thickness + LV wall thickness)^3^- (diastolic LV diameter)^3−^13.6.

Left ventricular mass was adjusted for the body surface area (BSA) using the following formula: BSA (m^2^) = 0.007184 x Height (cm)^0.725^ x Weight (kg)^0.425^.

### Carotid ultrasonography

Subjects enrolled into the study were investigated for the presence of atherosclerotic carotid plaques by B-mode using a 10-MHz linear transducer (Vingmed System Five, Norway). The subjects were examined in the supine position with the neck extended. Carotid intima-media thickness (CIMT) measurements were performed in the longitudinal plane at the point of maximum thickness on the wall of the common carotid artery along a 1 cm section of the artery proximal to the carotid bulb. Carotid plaque was defined as a thickness more than 1.5 mm as measured from the media–adventitia interface to the intima–lumen interface [19]. At least three measurements were done for each patient and the mean value of these measurements was used in the analysis.

### Brachial artery endothelial function

FMD measurements of the brachial artery were performed by an experienced cardiologist. The arms of patients were immobilized in the extended position, and brachial artery was scanned longitudinally 3 to 5 cm above the antecubital fossa. Baseline measurements of the brachial artery were recorded and then the cuff inflated to 200 mmHg (or 50 mmHg higher than systolic blood pressure) for 5 min to create forearm ischemia. Afterwards cuff was deflated and diameter of the brachial artery was measured at 60 s after deflation. FMD was defined as the percent change in the diameter of the brachial artery from baseline to post-reactive hyperemia. All measurements were performed by a single investigator blinded to clinical details.

### Flow cytometric analysis of CD133+ cells

For analysis of circulating CD133+ cell counts, blood samples were withdrawn before the midweek HD session and were processed immediately. After purification, 5 × 10^5^ cells were stained with anti-human CD133 phycoerythrin (PE) (MiltenyiBiotec GmbH, BergischGladbach, Germany), and PE conjugated isotype control (IC) monoclonal antibodies (mAbs) and incubated for 20 min at room temperature. Stained cells were fixed in 1% paraformaldehyde and analyzed with BD FACSCalibur with CellQuest Software (BD Bioscience, San Jose, CA). Cells were analyzed using gating strategies that conformed to International Society of Hematotherapy and Graft Engineering criteria and CD133+ cell counts were determined.

### Statistical analysis

Statistical Package for Social Sciences for Windows was used for the statistical analysis (SPSS Inc., Chicago, IL). Results were expressed as mean ± SD unless otherwise stated. Data with non-normal distribution were presented as median (25–75% interquartile range). Correlation coefficients and significance were calculated by Pearson’s test. Correlations between parameters with non-normal distribution were analyzed with Spearman’s test. Comparisons of continuous data for the groups were performed using the Student t-test or the Mann–Whitney U-test. Since CD133+ cell count and FMD were not normally distributed, logarithmic transformation (Log10) was applied to these variables. Independent variables that significantly correlated with the dependent variable in univaried analysis were included in the multiple linear regression analysis. Dialysis vintage, LVMI and LogCD133 counts were the independent variables entered into the multiple regression performed to predict LogFMD (model R = 0.57, adjusted R square = 0.27, p = 0.001). All tests of significance were two sided, and differences were considered statistically significant when the *P*-value was <0.05.

## Results

Baseline demographic and laboratory parameters of the patients were presented in Table 1. Patients were divided into 2 groups according to median CD133+ cell counts **(**Table 1). Serum TNF-α, leptin and resistin levels were found to be significantly higher in high CD133+ group however, FMD was significantly lower in high CD133 group compared to low CD133+ group.

**Table 1:** Baseline demographic and laboratory parameters of the patients (BMI: body mass index, LVH: left ventricular hypertrophy, LVMI: left ventricular mass index, CIMT: carotid intima-media thickness, FMD: flow mediated diltation, CRP: C-reactive protein, TNF-α: tumor necrosis factor-α, IL-6: interleukin-6)

Parameters	All patients (*n* = 58)	High CD133+ group	Low CD133+ group	*p* value ^a^
Age(years)	58 ± 15	56 ± 17	60 ± 12	0.30
Gender (M/F)	28/30	15/14	13/16	0.50
Dialysis intage (months)	81.6 ± 44.4	7.35 ± 3.92	6.50–3.55	0.40
Kt/V	1.88 ± 0.34	1.89 ± 0.31	1.87 ± 0.38	0.79
BMI (kg/m^2^)	25 ± 5	26 ± 4	24 ± 5	0.14
LVH (n,%)	19 (33%)	11 (38%)	8 (27%)	0.36
LVMI (g/m^2^)	114 ± 29	118 ± 28	109 ± 30	0.31
Carotid plaque (n,%)	25 (43%)	11 (37%)	14 (48%)	0.37
CIMT (mm)	0.87 ± 0.19	0.86 ± 0.17	0.88 ± 0.22	0.80
FMD (%)	6.41 (2.80–10.80)	3.46 (0.88–9.63)	8.50 (6.12–12.51)	0.02
Hemoglobin (g/dL)	11.88 ± 1.24	12.16 ± 1.40	11.61 ± 1.03	0.10
Albumin (g/dL)	4.09 ± 0.24	4.15 ± 0.24	4.03 ± 0.22	0.04
CRP (mg/dL)	0.74 (0.38–1.41)	0.70(0.38–1.20)	0.80(0.46–1.53)	0.73
TNF-α (pg/mL)	31.64(23.81–61.29)	54.51(28.92–70.08)	26.19(22.15–37.29)	0.002
IL-6 (pg/mL)	11.71 (8.22–21.12)	12.29(8.69–22.94)	11.31(5.82–17.81)	0.46
Leptin (ng/mL)	23.58(7.12–54.22)	32.37(12.74–72.29)	15.50(5.38–37.12)	0.03
Adiponectin(μg/mL)	30.42(20.45–45.11)	26(18.38–37.85)	31.81(22.82–45.25)	0.25
Resistin (ng/mL)	3.25(2.30–4.30)	3.92(3–5.13)	2.91(1.89–3.34)	<0.001
CD133+ cell count (%)	0.80(0.60–1.00)	1(0.85–1.27)	0.6(0.51–0.65)	<0.001

^a^Comparison of the results between high and low CD133+ groups

### CD133+ cell counts and CV paramaters

**Fig. 1:**
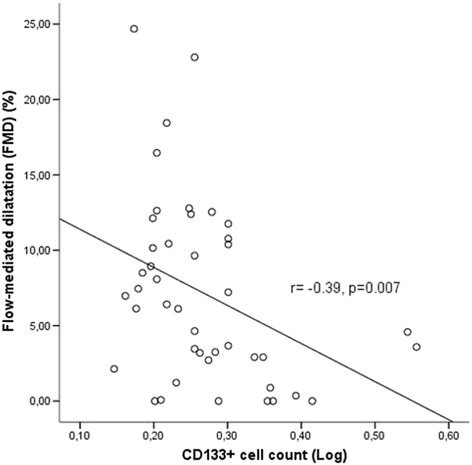
CD133+ cell count was significantly associated with FMD

CD133+ cell counts were significantly inversely associated with FMD (*r* = −0.39, *p* = 0.007) (Fig. 1). However CD133+ cell count was not related to CIMT (*r* = −0.06, *p* = 0.66).

### Adipocytokines and CD133+ cell counts

**Fig. 2:**
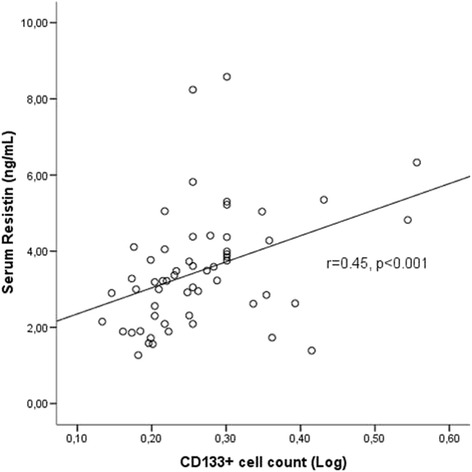
Serum resistin levels were positively correlated with CD133+ cell count

**Fig. 3:**
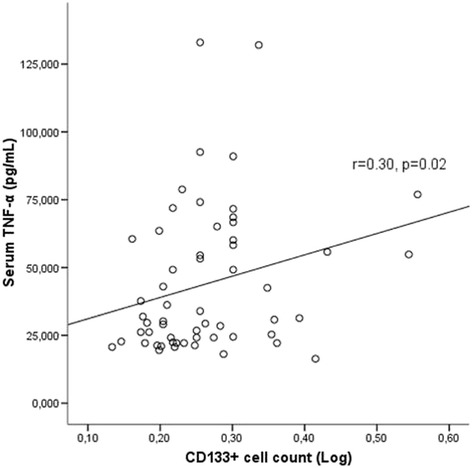
Serum TNF-α levels were positively correlated with CD133+ cell count

**Fig. 4:**
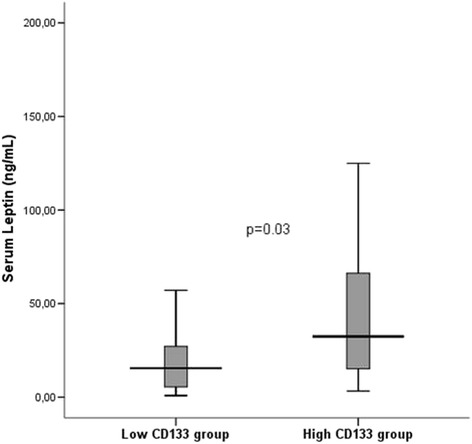
Serum leptin levels were higher in high CD133+ group compared to that of low CD133+ group

CD133+ cell counts were correlated with serum resistin levels (*r* = 0.45, *p* < 0.001) (Fig. 2) and serum TNF-α levels (*r* = 0.31, *p* = 0.02) (Fig. 3) in HD patients. When patients were divided into two groups according to median CD133+ cell count, serum leptin levels were found to be significantly higher in high CD133 group compared to low CD133 group [32.37(12.74–72.29) vs 15.50(5.38–37.12) ng/mL, *p* = 0.03] (Fig. 4). However in correlation analysis, no significant relationship was found between CD133+ cell count and serum leptin levels (r = 0.21, p = 0.12). CD133+ cell counts were not found to be associated with serum adiponectin levels (*r* = −0.04, *p* = 0.71).

**Fig. 5:**
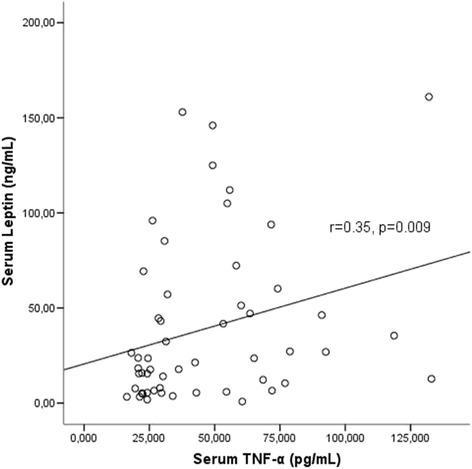
Serum leptin levels were correlated with serum TNF-α levels

**Fig. 6:**
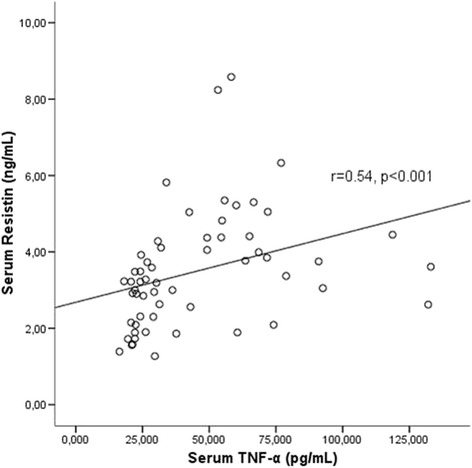
Serum resistin levels were positively correlated with serum TNF-α levels

Serum leptin levels were significantly higher in the female patients compared to male patients [32.37 (10.13–94.91) vs 15.69 (6.56–33.31), *p* = 0.04]. Serum IL-6, TNF-α, adiponectin and resistin levels were not different between male and female patients. Body mass index (BMI) was positively correlated with serum leptin levels (*r *= 0.68, *p* < 0.001) and negatively with serum adiponectin levels (*r*= −0.43, *p* = 0.001). Serum leptin levels were significantly correlated with TNF-α levels (*r* = 0.35, *p* = 0.009) (Fig. 5). Serum adiponectin levels were negatively correlated with serum leptin (*r *= −0.28, *p*= 0.03), uric acid (*r *= −0.30, *p* = 0.02) and triglyceride levels (*r*= −0.34, *p* = 0.007). Serum adiponectin levels were not related to inflammation namely, TNF-α (*r* = 0.05, *p* = 0.68), IL-6 (*r* = 0.19, *p* = 0.16) or resistin (*r* = −0.003, *p* = 0.98). Serum resistin levels were significantly associated with TNF-α (*r* = 0.54, *p* < 0.001) (Fig. 6), leptin (*r* = 0.29, *p* = 0.03) and uric acid levels (*r* = 0.31, *p* = 0.01).

### Adipocytokines and CV parameters

Serum IL-6 levels were significantly associated with LVMI (*r* = 0.31, *p* = 0.03). Serum IL-6 levels were significantly higher in patients with carotid plaque compared to patients without plaque [12.75(9.91–28.68) vs 8.27(5.97–14.04) pg/mL, *p* = 0.02]. Serum hsCRP levels were also higher in patients with carotid plaque compared to patients without plaque [0.90 (0.46–1.5) vs 0.53 (0.25–0.85) mg/dL, *p* = 0.02]. FMD was not associated with serum leptin (*r* = −0.03, *p* = 0.82), adiponectin (*r* = 0.13, *p* = 0.36), resistin (*r* = 0.21, *p* = 0.15), TNF-α (*r* = 0.21, *p* = 0.16) or IL-6 (*r* = −0.22, *p* = 0.16). CIMT was not found to be related to serum leptin (*r* = −0.09, *p* = 0.56), adiponectin (*r* = 0.09, *p* = 0.53), resistin (*r* = −0.24, *p* = 0.09), TNF-α (*r* = −0.20, *p* = 0.17) or IL-6 (*r* = 0.17, *p* = 0.25).

On comparing adipocytokine levels of patients with and without carotid plaque, there was no difference in serum leptin levels [19.82 (7.92–45.58) vs 35.45 (6.01–60.32) ng/mL, *p* = 0.58], adiponectin [26.66 (17.83–37.54) vs 33.95 (21.75–53)μg/mL, *p* = 0.24], resistin [3(1.99–3.79) vs 3.66 (2.83–4.38) ng/mL, *p* = 0.13] and TNF-α [30.78 (21.72–54.04) vs 38.45 (26.62–65.95) pg/mL, *p* = 0.11].

FMD was significantly negatively associated with LVMI (*r* = −0.44, *p* = 0.02) and dialysis vintage (*r* = −0.34, *p* = 0.02). LVMI was significantly related to dialysis duration (*r* = 0.38, *p* = 0.009). LVMI was found to be higher in patients with carotid atheroma plaque compared to patients without plaque (121 ± 27 vs 104 ± 27 g/m^2^, *p* = 0.03). CIMT was significantly associated with age (*r* = 0.36, *p* = 0.01).

### Multivariate analysis

In multiple linear regression analysis to determine the factors predicting LogFMD; dialysis vintage, LVMI and LogCD133+ cell counts were included as independent variables (*R* = 0.57, adjusted R square = 0.27, *p* = 0.001) (Table 2). CD133+ cell count and LVMI were found to significantly predict FMD (*p* = 0.03 and *p* = 0.04 respectively). Back-transformed unstandardized beta coefficient of LogCD133 in multiple linear regression model was found to be −23.80 (CI: 95%: lower bound: −411.20 and upper bound: -2.26).

**Table 2:** Multiple linear regression analysis to determine the factors predicting LogFMD (model *R* = 0.57, adjusted R square = 0.27, *p* = 0.001)

	β	Standardized β	CI (95%)		*P* value
			Lower bound	Upper bound	
Dialysis vintage (months)	−0.026	−0.24	−0.05	0.005	0.10
LVMI (g/m^2^)	−0.004	−0.29	−0.008	0.001	0.04
CD133+ cell count (Log)	−1.358	−0.29	−2.613	−0.102	0.03

## Discussion

In this study, CD133+ cell counts were found to be independently associated with endothelial dysfunction in HD patients. Although circulating progenitor cells such as CD133+ cells are known to have pro-angiogenic and repair properties in various tissues, they may also be associated with increased inflammation and atherosclerotic plaque instability by promoting neovascularization in the plaques leading to the progression of atherosclerosis by enhancing the entry of inflammatory cells and cytokines into the arterial wall [6–10]. In the study by Sepp et al. [7], the number of CD133+ cell counts was similar in subjects with stable and unstable carotid plaques however CXCR4 expression of CD133+ cells were found to be associated with plaque instability in patients with carotid artery stenosis. In the study by Bartunek et al. [9], intracoronary injection of CD133+ cells was found to increase in-stent restenosis and progression of atherosclerosis. In another study, the number of circulating progenitor cells (CD45+ CD34+ CD133+) was inversely associated with FMD in patients with COPD [10]. In the present study, for the first time in the literature, we found that increased CD133+ cell counts were associated with endothelial dysfunction in HD patients. However no relationship was found between CD133+ cell counts and carotid atherosclerosis. These findings suggested that CD133+ cells might play role in endothelial dysfunction which is early stage of atherosclerosis rather than the already advanced atherosclerosis.

In a recent study performed on HD patients by Lineen et al. [11], the relationships between CD133+ cells and LVMI were investigated. CD133+ cell count were not found to be associated with LVMI in parallel to our findings. Hypervolemia and anemia are known to be the strong factors in the pathogenesis of LVH in HD patients, thus the role of stem cells in the remodelling of myocardium may be outweighed by these factors.

CD133+ cells have been recently found to be related to inflammation. In the study by Burger et al. [6], human umbilical cord blood derived CD133+ cell administration exacerbated ischemic acute kidney injury and inflammation in a mice model. Increased neutrophil infiltration and myeloperoxidase activity were observed in kidneys and plasma TNF-α levels were also increased. Authors suggested that TNF-α may be responsible for the pro-inflammatory effects of CD133+ cells. Consistent with this result, we also reported similar correlation between CD133+ cell counts and serum TNF-α levels in HD patients. CD133+ cell counts were also correlated with serum resistin known as an important novel pro-inflammatory adipocytokine. In addition, serum leptin levels were related to higher CD133+ cell counts. These relationships of CD133+ cell count with the adipocytokine levels have not been previously reported in HD patients. All these findings suggest an active role of adipose tissue and adipocytokines in regulation of CD133+ cell counts.

Adipocytes produce a wide range of protein signals and factors termed adipocytokines which include TNF-α, IL-6, leptin, adiponectin, resistin, plasminogen activator inhibitor, angiotensinogen, monocyte chemotactic protein-1, macrophage migration inhibitory factor and vascular endothelial growth factor [20]. They serve as the signals for the effects of adipocytes on insulin resistance, inflammation, dyslipidemia, hypertension, and atherosclerosis [12–15]. In this study, serum IL-6 levels seemed to be associated with left ventricular hypertrophy and presence of atherosclerotic carotid plaques. Adipocytokines such as leptin and resistin were associated with inflammation but they did not seem to be directly related to parameters of endothelial dysfunction and atherosclerosis in HD patients.

Serum leptin levels are known to be correlated with body fat mass and adiponectin is known to be inversely associated with BMI in the general and dialysis populations [21, 22]. Our study was also in agreement with these studies that serum leptin and adiponectin levels were both significantly associated with BMI in opposite directions. Leptin is known to promote vascular inflammation and oxidative stress [15, 23, 24]. Leptin directly stimulates the production of acute-phase proteins in liver cells [25]. Furthermore, a significant correlation between leptin and CRP concentrations was demonstrated in CKD patients [16]. In the study by Pecoits-Filho et al. [21] performed on patients with end-stage renal disease, a positive correlation was found between IL-6 and serum leptin concentrations. In the present study, we also observed that serum leptin levels were significantly associated with serum TNF-α levels which underlines the relationship between leptin and inflammation. In a study performed on obese individuals, serum leptin levels were independently associated with CIMT [26]. However we did not observe a relationship between serum leptin levels with CIMT in HD patients. Role of leptin on atherosclerosis might be overriden by other uremia-related CV risk factors.

Resistin is a novel adipocytokine that may play a role in the pathophysiology of insulin resistance and obesity [27]. Resistin is also known to increase inflammation by upregulating IL-6 and TNF-α levels [18, 28, 29]. In aggrement with these results, serum resistin levels were significantly associated with pro-inflammatory cytokines such as serum TNF-α and leptin levels in the present study. Furthermore, we also reported a significant association of serum resistin levels with CD133+ cell counts in HD patients. Resistin has been previously reported to play a role in endothelial dysfunction and atherosclerosis in non-uremic population [18, 30]. However, serum resistin levels were not found to be related to FMD and CIMT in our study. Lack of association of resistin with endothelial dysfunction and atherosclerosis might be due to the presence of other more dominant uremia-related toxins leading to increased cardiovascular risks.

## Conclusions

Cross-sectional nature of the study, relatively small sample size, lack of control group are the limitations of the study. Possible significant relationships between CD133+ cell counts and atherosclerosis (namely CIMT) might not be found due to relatively small sample size. However investigation of multiple parameters of adipocytokines and cardiovascular risk factors and novel findings are the strengths of the study.

In conclusion, CD133+ cells were associated with inflammation and endothelial dysfunction in HD patients. Serum leptin, resistin and TNF-α levels were positively related to CD133+ cell count. Impaired regulation of undifferentiated stem cells and adipocytokines might contribute to endothelial dysfunction in HD patients.

## Abbreviations

BSA: Body surface area
CIMT: Carotid intima-media thickness
CKD: Chronic kidney disease
ELISA: Enzyme-linked immunoadsorbent assays
FMD: Flow-mediated dilatation
HD: Hemodialysis
hsCRP: High sensitive C-reactive protein
IL-6: Interleukin-6
LVH: Left ventricular hypertrophy
LVMI: Left ventricular mass index
PE: Phycoerythrin
TNF-α: Tumor necrosis factor-α
